# Retinochoroidal microvascular changes in newly developed obese children: an optical coherence tomography angiography study

**DOI:** 10.1186/s12886-022-02664-9

**Published:** 2022-11-17

**Authors:** Shu Han, Zhenhua Leng, Xiaoxiao Li, Wen Yan, Shiya Shen, Lei Liu, Hui Zhu, Dan Huang, Hu Liu

**Affiliations:** 1 Department of Ophthalmology, Children’s Hospital of Soochow University, Suzhou, China; 2 Department of Ophthalmology, The First Affiliated Hospital With Nanjing Medical University, 300 Guangzhou Road, Nanjing, 210029 China; 3 Department of Ophthalmology, The Fourth Affiliated Hospital of Nantong University (Yancheng Children’s Hospital), Yancheng, China; 4 School of Medical Technology, Jiangsu College of Nursing, Huai’an, China; 5 Department of Child Healthcare, The First Affiliated Hospital With Nanjing Medical University, Nanjing, China

**Keywords:** Newly developed obese children, Choriocapillaris, Optical coherence tomography angiography, Retinal thickness, Vessel density

## Abstract

**Background:**

Childhood obesity was associated with retinochoroidal microvascular changes using optical coherence tomography angiography (OCTA), but obesity duration was neglected. Obesity is chronic and progressive and obesity duration is related to microvascular function. Thus, it is important to identify microvascular changes in obese children timely to allow possible interventions with the increase in the number of obese children. This pilot study aimed to characterize retinochoroidal microvascular changes in newly developed obese children compared to age- and sex-matched controls.

**Methods:**

Forty newly developed obese children and 40 age- and sex-matched controls were recruited. All subjects completed comprehensive eye examinations, including axial length, cycloplegic refraction, optical coherence tomography angiography scans (AngioVue; Optovue Inc), etc.

**Results:**

There were no statistically significant differences between groups in terms of month age (*P* = 0.927), spherical equivalent refraction (*P* = 0.753) and axial length (*P* = 0.196). Newly developed obese children had lower vessel density (VD) in the inferior parafovea (*P* = 0.026), nasal parafovea (*P* = 0.038) and temporal perifovea (*P* = 0.026) of deep vascular complex (DVC), higher VD in the fovea of superficial vascular complex (*P* = 0.021) and the fovea of DVC (*P* = 0.016), and smaller foveal avascular zone (*P* = 0.003) when compared to controls. Also, no apparent differences in any quadrant of total retinal thickness, subfoveal choroidal thickness (SFCT), and choriocapillaries fow voids were detected (all *P* > 0.05).

**Conclusion:**

Retinochoroidal microvascular changes had already occurred in newly developed obese children, so early screening and close follow-up eye examinations were recommended; Retinal microvascular insult may precede its structural change and that retina may be a better candidate to predict the onset of retinochoroidal microvascular changes than choroid in obese children.

## Introduction background

With an increasing global prevalence of unhealthy and sedentary lifestyles, childhood obesity has become a major worldwide health problem [1, 2]. The prevalence of childhood obesity is still increasing despite potential preventive measures [3]. In addition, the impact of childhood obesity on health is widespread [4, 5]. As we all know, childhood obesity is associated with an increased risk of hypertension, type 2 diabetes and coronary heart disease in later life [6, 7], and the underlying pathology in these diseases occurs at the level of the microvasculature.

Studies have reported that childhood obesity was associated with retinochoroidal microvascular changes using optical coherence tomography angiography (OCTA) [8–10]. However, it is unclear whether estimated associations are mainly driven by persons with long-standing obesity, persons with recently developed obesity, or some combination of both. The adolescents in these studies may have a long duration of obesity. Moreover, obesity is chronic and progressive [11, 12], and the association between the duration of obesity and microvascular function has been reported [13]. In addition, for young obese children or those with short disease duration, fundus examination has not been officially recommended for now. Thus, it is important to identify microvascular changes in obese children timely to allow possible interventions with the increase in the number of obese children.

This study was designed to evaluate retinochoroidal microvascular changes in newly developed obese children using OCTA, thereby providing data and reference information for the prevention and treatment of micro-vasculopathy in children with obesity.

## Subjects and methods

### Study population

This cross-sectional study was conducted from July 2020 to September 2021 at the First Affiliated Hospital with Nanjing Medical University. In concordance with the tenets of the Declaration of Helsinki, informed consent was obtained from all parents or legal guardians. This study was approved by the Ethics Committee of The First Affiliated Hospital with Nanjing Medical University.

### Inclusion and exclusion criteria

Forty newly developed obese children aged 84–96 months and an equal number of age- and sex-matched healthy control subjects were recruited. Obesity was defined as the body mass index (BMI) greater than or equal to the 95th percentile [14], according to Chinese age- and sex- specific BMI reference values [15]. Children, who were diagnosed obesity in the last three years, were classified as newly developed obesity. All included subjects were required to have 20/20 or better best-corrected visual acuity (BCVA).

The exclusion criteria were the presence of amblyopia, nystagmus, retinal disease, hypermetropia exceeding 2 D, myopia and astigmatism exceeding 1 D, glaucoma, intraocular inflammation, strabismus, elevated intraocular pressure > 21 mmHg and media opacity, such as corneal disease or cataract. Patients with a history of prematurity, neurologic disease, ocular trauma, ocular surgery or systemic conditions that could alter the microvasculature, such as diabetes, hypertension, cardiovascular disease, renal disease, and autoimmune disease, were excluded. Additionally, patients with head, neck or other injuries preventing proper positioning or unable to maintain retinal fixation on a specified target (owing to poor cooperation) were excluded.

### BMI and Anthropometric Measurements

Body weight was measured the children having fasted for 12 h, having emptied the bladder, and standing in light clothing and without shoes and height was measured using a standard scale weighing machine. Height and weight were measured to the nearest 0.1 cm and 0.1 kg, respectively. BMI was calculated as weight divided by height squared (in kg/m^2^). We collected detailed information regarding previous height and weight through medical records for each child.

### Ocular examinations

Ophthalmological examinations were performed, including distance visual acuity (VA), refraction diopter after cycloplegia, axial length (AL) measurements, OCTA (AngioVue; Optovue, Inc., Fremont, California USA), etc.

Distance VA was measured with the Early Treatment Diabetic Retinopathy Study (ETDRS) visual acuity chart (Precision, Vision, LaSalle, IL, USA) at a distance of 4 m. BCVA was recorded with full correction under cycloplegia. The refractive status of each participant was measured after cycloplegia using table-mounted autorefraction (Cannon R-F10, Tokyo, Japan). AL measurement was performed with an IOL Master (Carl Zeiss Meditec, Jena, Germany; V5.5.0.0062).

OCTA was used to capture the OCTA scanning for microvascular in the macular retina and choriocapillaris (CC). The AngioVue OCTA has an A-scan rate of 70,000 scans per second, and 2 successive B-scans were taken at the same location.

Each imaging cube consisted of 2 repeated volumes (304 B-scans × 304 A-scans). 6 mm × 6 mm macular scans centered on the fovea were performed. Retinal layers were automatically segmented by the AngioVue software. Superficial vascular complex (SVC) extends from the internal limiting membrane to 10 μm above the lower boundary of the inner plexiform layer (IPL). Deep vascular complex (DVC) is measured from 10 μm above the lower boundary of the inner plexiform layer (IPL) to 10 μm below the lower boundary of the outer plexiform layer (OPL). The vessel density (VD) was defined as the proportion of vessel area showing blood flow relative to the total image area. Following the ETDRS, the images were made in the nine subfields: foveal, parafoveal and perifoveal quadrants (superior, inferior, nasal and temporal) [15]. The AngioVue software automatically analyzed the VD and retinal thickness in each quadrant.

As described before [16, 17], we imported the OCTA images into MATLAB (MathWorks, Inc., Natick, Massachusetts) and segmented the choriocapillaris slabs (31 μm to 59 μm below the retinal pigment epithelium). Then the retinal vessel projection artifacts were removed from the CC. The area of CC flow voids was defined as a percentage between the region absent from flow and the total scanned region. Finally, the binarized image of CC was imported into Image J software (National Institutes of Health, Bethesda, MD) for calculating the size and number of flow voids. The subfoveal choroidal thickness (SFCT) extends from the outer border of the retinal pigment epithelium to the inner border of the sclera running through the center of the fovea. And it was measured by a trained examiner using a manual caliper of the device software. All scans were performed in dim light by the same technician trained in the use of the equipment. Poor-quality scans with a signal strength lower than 7 were excluded from the analysis.

### Statistical analyses

Retinal parameters (retinal thickness, VD and foveal avascular zone), SFCT and CC flow void from right eyes were included for statistical analysis. The Kolmogorov–Smirnov test was used to evaluate the normal distribution. According to normality tests, either independent samples t-test or Mann–Whitney U-test was used to compare differences between obese children and control. The Chi-square test was used to compare percentages between groups. All statistical tests were performed using the Statistical Package for the Social Sciences (SPSS) statistical software (V.13.0, IBM, USA), and two-sided *P* < 0.05 was considered statistically significant. Regarding refraction, the spherical equivalent was calculated as a spherical diopter plus half of the diopter of cylindrical power using cycloplegic refraction.

## Results

Forty newly developed obese children with a mean month age of 90.13 ± 3.74 (range, 84–96 months) and 40 age- and sex-matched controls with a mean month age of 90.13 ± 3.55 (range, 84–96 months) are shown in Table 1. No significant differences were found in the month age (*P* = 0.927), spherical equivalent refraction (*P* = 0.753), and AL (*P* = 0.196) of the two groups. As expected, weight, height, and BMI were significantly higher in obese children than in controls (all *P* < 0.05).

**Table 1:** Anthropometric and ocular characteristics of newly developed obese children and control subjects

Variables	obesity	control	*P*
Month age	90.13 ± 3.74	90.13 ± 3.55	0.927^b^
Height, cm	131.38 ± 8.76	128.67 ± 5.43	0.015^b^
Weight, kg	38.79 ± 6.02	26.00 ± 2.33	< 0.001^b^
BMI (kg/m^2^)	22.34 ± 1.64	15.67 ± 0.40	< 0.001^a^
AL (mm)	23.20 ± 0.76	22.98 ± 0.71	0.196^a^
SE(D)	1.03 ± 0.51	1.07 ± 0.55	0.753^a^

*BMI* Body mass index, *SE* Spherical equivalent, *AL* Axial length

^a^t test

^b^Mann-Whitney U test

Quantitative total retinal thickness, VD, and foveal avascular zone measurements based on the ETDRS grid from 6 mm × 6 mm macular scans were measured. The total retinal thickness measurements in newly developed obese children and controls are shown in Table 2. No statistically significant difference was found between the two groups in any quadrant of the total macular thickness (all *P* > 0.05).

**Table 2:** Total retinal thickness in newly developed obese children and control subjects

Variables	Obesity	Control	*P*
ILM to RPE (total retina) thickness, µm			
Average	290.62 ± 12.44	291.35 ± 12.23	0.793^a^
CSF	239.12 ± 13.74	232.47 ± 18.01	0.067^a^
SI	323.02 ± 12.53	322.32 ± 13.43	0.809^a^
II	319.63 ± 15.23	316.44 ± 13.67	0.328^a^
NI	321.52 ± 13.13	318.09 ± 13.22	0.248^a^
TI	310.28 ± 13.71	309.27 ± 12.58	0.733^a^
SO	286.92 ± 12.14	289.71 ± 12.52	0.199^b^
IO	275.23 ± 13.86	276.76 ± 13.73	0.621^a^
NO	301.66 ± 13.43	303.20 ± 14.92	0.628^a^
TO	272.88 ± 14.17	274.51 ± 12.89	0.591^a^

*ILM* Internal limiting membrane, *RPE* Retinal pigment epithelium, *CSF* Central fovea; *SI* Superior inner ring, *II* Inferior inner ring, *NI* Nasal inner ring, *TI* Temporal inner ring, *SO* Superior outer ring, *IO* Inferior outer ring, *NO* Nasal outer ring, *TO* Temporal outer ring

^a^t test

^b^Mann-Whitney U test

In addition, newly developed obese children had higher VD in the fovea of SVC (24.44 ± 6.44 VS 21.02 ± 6.55, *P* = 0.021) and the fovea of DVC (38.86 ± 6.96 VS 35.00 ± 7.03, *P* = 0.016) when compared to controls (Table 3). Lower VD in the inferior parafovea (54.54 ± 4.80 VS 56.80 ± 4.04, *P* = 0.026), nasal parafovea (57.14 ± 3.72 VS 58.96 ± 3.99, *P* = 0.038) and temporal perifovea (54.96 ± 3.75 VS 56.86 ± 3.76, *P* = 0.026) of DVC were found in newly developed obese children when compared to controls. VD was not significantly different between controls and obese children in any quadrant of SVC and remaining regions of DVC (all *P* > 0.05). Additionally, a considerably smaller foveal avascular zone was found in newly developed obese children (*P* = 0.003).

**Table 3:** Macular vessel density and foveal avascular zone parameters in newly developed obese children and control subjects

Retinal region	Obesity	Control	*P*
SVC			
Average	50.37 ± 2.95	50.88 ± 2.33	0.389^a^
Fovea	24.44 ± 6.44	21.02 ± 6.55	0.021^a^
SI	51.96 ± 4.53	53.18 ± 4.03	0.235^b^
II	50.66 ± 4.87	52.35 ± 4.69	0.080^b^
NI	51.27 ± 4.27	51.38 ± 3.67	0.977^b^
TI	51.21 ± 4.43	52.03 ± 3.27	0.758^b^
SO	51.22 ± 3.14	51.85 ± 2.32	0.308^a^
IO	51.53 ± 3.23	51.90 ± 2.59	0.522^b^
NO	54.23 ± 2.68	54.85 ± 2.10	0.250^a^
TO	47.31 ± 2.94	47.82 ± 2.77	0.427^a^
DVC			
Average	52.84 ± 4.60	54.61 ± 4.41	0.083^a^
Fovea	38.86 ± 6.96	35.00 ± 7.03	0.016^a^
SI	56.68 ± 4.32	58.30 ± 4.18	0.093^a^
II	54.54 ± 4.80	56.80 ± 4.04	0.026^b^
NI	57.14 ± 3.72	58.96 ± 3.99	0.038^a^
TI	56.61 ± 4.05	58.19 ± 3.27	0.090^b^
SO	52.05 ± 6.05	54.38 ± 4.86	0.063^b^
IO	51.72 ± 5.21	53.56 ± 5.77	0.137^a^
NO	50.70 ± 5.95	52.43 ± 5.98	0.108^b^
TO	54.96 ± 3.75	56.86 ± 3.76	0.026^a^
FAZ	0.25 ± 0.09	0.32 ± 0.10	0.003^b^

*SVC* Superficial vascular complex, *DVC* Deep vascular complex, *FAZ* Foveal avascular zone, *CSF* Central fovea, *SI* Superior inner ring, *II* Inferior inner ring, *NI* Nasal inner ring, *TI* Temporal inner ring, *SO* Superior outer ring, *IO* Inferior outer ring, *NO* Nasal outer ring, *TO* Temporal outer ring

^a^t test

^b^Mann-Whitney U test

There was no significant difference in the subfoveal choroidal thickness (*P* = 0.903), areas of fow voids (*P* = 0.126), size of fow voids (*P* = 0.148) and the number of fow voids (*P* = 0.335) between the two groups (Table 4).

**Table 4:** SFCT and choriocapillaries fow voids in newly developed obese children and control subjects

	Obesity	Control	*P*
SFCT	301.33 ± 42.49	299.79 ± 66.82	0.903^a^
Choriocapillaries fow voids			
Areas of fow voids, %	14.72 ± 0.37	14.59 ± 0.43	0.126^a^
Size of fow voids, pixels	10.62 ± 0.82	10.37 ± 0.66	0.148^a^
Number of fow voids	2228.83 ± 146.13	2256.30 ± 103.19	0.335^a^

*SFCT* Subfoveal choroidal thickness

^a^t test

## Discussion

In the present study, VD of the retina in newly developed obese children was significantly different from those in the control group, especially in DVC, and no significant difference was found in total retinal thickness, the subfoveal choroidal thickness or choriocapillaries fow voids. Our findings suggested retinochoroidal microvascular changes had already occurred in newly developed obese children, so early screening and close follow-up eye examinations were recommended. Additionally, retinal microvascular insult may precede its structural change and that retina may be better candidate to predict the onset of retinochoroidal microvascular changes than choroid in the early stages of childhood obesity, which may help to characterize the underlying pathophysiology of obesity and enable early detection and prevention of obesity-related ocular complications.

Unlike previous studies, we only included newly developed obese children instead of those with long-standing obesity to focus on the retinochoroidal microvascular in the early stage of childhood obesity. In order to deduce more accurate results, we only analyzed OCTA data from the right eyes and considered AL measurements.

No statistically-significant difference in total retinal thickness was found in obese children compared to controls, consistent with previous studies [18]. However, Ersan et al. found a significant thinner macular thickness, and Kurtul et al. found a higher FRT in obese children compared to nonobese children, contradicting the results of our study [9, 19]. Besides the difference in ethnicity, inclusion criteria, and imaging device, younger newly developed obese children in the present study should be mentioned, which will be discussed in details later.

In agreement with the previous study, newly developed obese children had lower VD in the inferior parafovea, nasal parafovea and temporal perifovea of DVC compared to the controls [20]. Additionally, our study found a significant increase in VD instead of a decrease in the fovea of SVC and fovea of DVC and foveal avascular zone was decreased in newly developed obese children. Although Can et al. advocated that the increased VD in the parafoveal region of the SCP may be related to the excessive proinflammatory cytokines, [8] the present study was limited by the lack of biochemical blood parameters. We speculated that endothelial dysfunction and oxygen deficit in obese children caused destruction and reduction of local capillaries, which could lead to compensatory dilation of local retinal capillaries [21–23]. Therefore, newly developed obese children had relatively higher VD in the fovea of the retina and a smaller foveal avascular zone.

Newly developed obese children had significantly different VD of the retina, especially in DVC. Consistent with our study, some studies have reported that the deep capillary plexus, the major part of DVC, may be more vulnerable to hypoxic retinal injury due to its more distal location from the retinal arterial and choroidal circulations and the higher metabolic demand in the middle retinal layers [24, 25]. Meanwhile, the possible explanation is that the distribution of retinal blood vessel varies with regions of retinal, leading to uneven microvascular changes in the retina. The pathophysiological mechanisms underlying these associations are unclear. Of note, monitoring the retinal VD of obese children may be necessary. Furthermore, no significant difference was found in any quadrant of total retinal thickness. These results proved that retinal microvascular abnormalities might precede retinal structural changes in obese children. But these hypotheses require additional longitudinal study.

In addition, there was no significant difference in the subfoveal choroidal thickness and choriocapillaries fow voids between the two groups, similar to the previous study [18]. However, several studies have reported choroid was thinner in obese children or adults compared to normal subjects [26, 27]. Celik et al. found an increase in subfoveal choroidal thickness in obese children. [10] Younger newly developed obese children in our study may be a reason for it. Obesity is chronic and progressive [11, 12], believed to start many years before manifested microvascular dysfunction occurs. Moreover, the association between the duration of obesity and microvascular function has been reported [13]. The younger newly developed obese children have a relatively shorter disease duration. It is possible that, in the present study, the duration of obesity was insufficient to cause the significant change of choriocapillaris measurements in obese children compared to controls. With the progress of obesity, microvascular destruction may be expected to worsen, resulting in substantial choriocapillaris changes. Therefore, VD of the retina may be more valuable to predict the onset of retinochoroidal microvascular changes than choroid in obese children in future cohort studies. Additionally, the difference may be due to variations in subjects' age, ethnicity, or imaging device.

To the best of our knowledge, this is the first study investigating retinochoroidal microvascular changes in newly developed obese children using OCTA. Strengths of our study include a sample of newly developed obese children instead of those with long-standing obesity to evaluate early obesity-induced retinochoroidal microvascular changes. However, the present study is limited by the lack of biochemical blood parameters in newly developed obese children and the control group. Besides, our conclusion may not be generalized because of the narrow age range. As noted, longitudinal studies in larger cohorts are needed to study retinochoroidal microvascular changes in obese children.

In conclusion, retinochoroidal microvascular changes had already occurred in newly developed obese children, so early screening and close follow-up eye examinations were recommended. Future longitudinal studies investigating OCTA findings are still needed to examine the progression of retinochoroidal microvascular changes in obese children.

## Data Availability

All data included in this study are available from the corresponding author upon reasonable request.

## Abbreviations

OCTA: Optical coherence tomography angiography
BMI: Body mass index
BCVA: Best-corrected visual acuity
VA: Visual acuity
AL: Axial length
CC: Choriocapillaris
SVC: Superficial vascular complex
IPL: Inner plexiform layer
DVC: Deep vascular complex
OPL: Outer plexiform layer
VD: Vessel density
SFCT: Subfoveal choroidal thickness
SPSS: Statistical Package for the Social Sciences
